# Effect of *Withania somnifera (L.) Dunal* on Sex Hormone and Gonadotropin Levels in Addicted Male Rats

**DOI:** 10.22074/ijfs.2016.4915

**Published:** 2016-06-01

**Authors:** Batool Rahmati, Mohammad Hassan Ghosian Moghaddam, Mohsen Khalili, Ehsan Enayati, Maryam Maleki, Saeedeh Rezaeei

**Affiliations:** 1 Neurophysiology Research Center, Shahed University, Tehran, Iran; 2 Department of Biochemistry, Faculty of Medicine, Shahed University, Tehran, Iran; 3 Department of Physiology, Faculty of Medicine, Shahed University, Tehran, Iran

**Keywords:** Morphine, Withania somnifera, Gonadotropins, Estrogen, Testosterone

## Abstract

**Background:**

Opioid consumption has been widely increasing across the globe; how- ever, it can cause adverse effects on the body. Morphine, an opioid, can reduce sex hor- mones and fertility. *Withania somnifera* (WS) is a traditional herb used to improve sexual activities. This study strives to investigate the effect of WS on sex hormones and gonado- tropins in addicted male rats.

**Materials and Methods:**

In this experimental study, forty-eight male National Maritime Research Institute (NMRI) rats were randomly divided into four groups: i. Control group, ii. WS-treated control group, iii. Addicted group, and iv. WS-treated addicted group. Wa- ter-soluble morphine was given to rats for 21 days to induce addiction, concurrently the treated groups (2 and 4) also received WS plant-mixed pelleted food (6.25%). At the end of the treatment, the sex hormone and gonadotropin levels of the rats’ sera were deter- mined in all the groups.

**Results:**

Except for follicle-stimulating hormone (FSH), morphine reduced most of the gonadotropin and sex hormone levels. Whereas WS caused a considerable increase in the hormones in the treated addicted group, there was only a slight increase in the treated control group.

**Conclusion:**

WS increased sex hormones and gonadotropins-especially testosterone, es- trogen, and luteinizing hormone-in the addicted male rats and even increased the proges- terone level, a stimulant of most sex hormones in addicted male rats.

## Introduction

Recent years have witnessed a rise in the use of opioids and the concomitant increase in their adverse effects such as addiction. 

Morphine, one of the strongest opioids, is routinely used to alleviate acute and chronic pain (1). However, it is accompanied by numerous side effects such as peripheral edema, immune suppression, hyperalgesia, sleep apnea, and complications in the digestive, nervous, and genitourinary systems (2,3). Morphine can adversely affect sexual hormones and fertility in humankind. Laboratory studies have also shown the side effects of this opioid on the sexual hormones of male and female rats (4). Decreased libido, increased rates of still birth, and genetic defects are some of the side effects of morphine use in rats (5). Studies on humans also suggest an inhibitory effect of endogenous opioids on testicular function and testosterone production (6). 

Chronic use of Morphine can cause not only hormonal and sexual disorders but also behavioral disorders in humans (7). Use of chemical drugs to treat these disorders has had an insignificant positive effect and numerous complications. According to the World Health Organization (WHO), about three-quarters of the world population relies upon traditional remedies (mainly herbs) for the health care of its people (8). Scientists, therefore, seek to identify new drugs among herbal medicines that would have fewer complications. 

*Withania somnifera* (WS), also known as ashwagandha and winter cherry, has been an important traditional herbal medicine for over 3000 years (9). WS is a densely pubescent shrub up to 1-m tall belonging to the family of Solanaceae. Chemical compounds including large quantities of antioxidants, tannis, flavonoids, and phenolic compounds have been identified in WS (10). It has been reported that WS possesses anti-inflammatory and antioxidant effects and can be useful in the treatment of endocrine and neural disorders (11). 

The medicinal properties of WS are attributed to specific secondary metabolites such as alkaloids and withasteroids-withanolides (12). WS produces the largest number of withanolides in diversified functional groups, some of which possess specific therapeutic significance (13). It has been shown that withanolide A may induce neurite development and be useful in the treatment of Alzheimer's disease, Parkinson’s disease, and other neurological disorders (14). 

One study demonstrated the positive effects of WS on male rats insofar as it raised the luteinizing hormone (LH) level in adult male rats (15). 

Studies also indicate that the aqueous extract of WS induces some changes in hypophyseal gonadotropins accompanied by an increase in folliculogenesis in immature female rats (16). Another study reported that WS increased libido in adult rats (17). A study on 150 adult men showed that WS treatment led to an increase in testosterone and LH and a decrease in follicle-stimulating hormone (FSH) and serum prolactin. The same study also reported that WS increased antioxidants and decreased oxidative agents, thereby reducing oxidative stresses (18). 

With regard to the effect of WS on sexual hormones and gonadotropins and sexual disorders in opioid users, this study sought to investigate the effect of this herb on sexual hormones and gonadotropins in addicted male rats. 

## Materials and Methods

### Experimental animals

In this experimental study, 48 male National Maritime Research Institute (NMRI) rats (Razi Institute, Iran) weighing an average of 250 g were randomly divided in to four groups: i. Control group, ii. WS-treated control group, iii. Addicted group, and iv. WS-treated addicted group. The rats were kept in cages at a temperature of 24 ± 2°C and were given access to food and water. Twelvehour light-dark cycling was set. 

### Study protocol

In order to induce morphine addiction in groups 3 and 4, water-soluble morphine was given for 21 days at the same time the treated groups (2 and 4) also received WS plant-mixed pelleted food. 

The morphine solution was given to rats in doses of 0.1, 0.2, and 0.3 mg/mL; each dose was administered for 48 hours and then a dose of 4 mg/mL was given for the remaining 15 days. Also, 3% sucrose was added to the solution to omit the bitterness of morphine. Addiction was verified by injecting 2 mg/kg of naloxone intraperitoneally and observing withdrawal symptoms (19). 

The methods were confirmed by the Ethics Committee of Shahid Beheshti University of Medical Sciences, and the laboratory animals were afforded due care in accordance with the regulations of the Committee for the Purpose of Control and Supervision on Experiments on Animals (CPCSEA). 

### Preparation of Withania somnifera

WS was obtained and approved for the study by the Department of Botany, Shahid Beheshti University of Medical Sciences. Its roots were separated and ground and then combined with pelleted food at a weight ratio of 6.25%. For this purpose, 1000 g of ground rat chow pellet was macerated with 62.5 g of dried WS powder. Next, the new miscellaneous compound was subjected to a pellet-maker device for a new mixed-pellet production. The treated groups (2 and 4) received WS mixed pelleted food for 21 days (20). Given a daily pellet weight consumption (5 /kg/d) in the rats and 6.25% WS in the new pellet, each animal received 0.3 ± 0.01 g/kg WS in each day. 

### Blood sampling

At the end of treatment, blood samples (3-5 cc) were obtained from the heart of all the rats. The sera were separated via centrifuge (Sigma 4-10, USA) at 2000 rpm for 15 minutes and stored at -70°C in a freezer for hormone analysis. 

### Plasma analysis

The plasma levels of the sex hormones and gonadotropins were verified using kits manufactured by Monobind Inc., USA, as well as Elisa kits (Labsystems Ltd., Finland). The measurements were done in the central laboratory of Shahid Mostafa Khomeini Hospital. 

### Statistical analysis

The data were analyzed with the one-way analysis of variance, followed by post-hoc Tukey test. A P<0.05 was considered statistically significant. 

### Results

**Fig.1:**
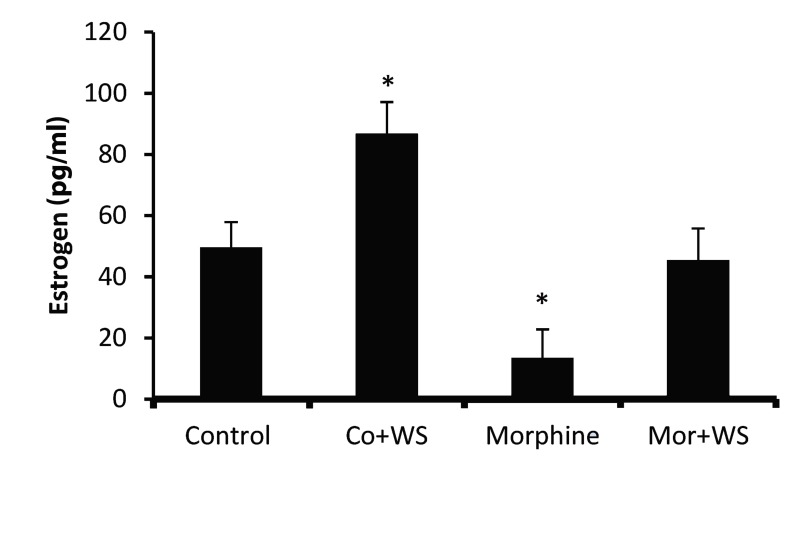
: The effect of *Withania somnifera* root on estrogen levels in control and morphine addicted groups. The data are expressed as mean ± SD. ^*^; P<0.05 as compared with the control group and morphine addicted groups respectively.

As is shown in Figure 1, morphine addiction reduced the estrogen level (22.76 pg/mL) compared to the control group (50.56 pg/mL). Whereas WS treatment in the control group caused a significant increase (P<0.05) in the estrogen level (86.89 pg/ mL), it had no significant effect on the estrogen level of the addicted male rats. 

**Fig.2:**
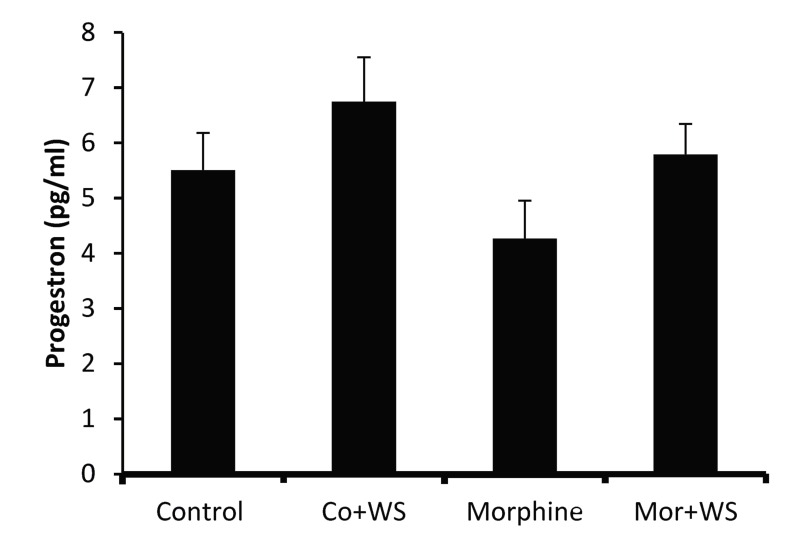
The effect of *Withania somnifera* root on progesterone levels in control and morphine addicted groups. The data are ex- pressed as mean ± SD.

The progesterone levels in all the experimental groups are illustrated in Figure 2. The progesterone level was increased in the WS-treated group (66.28 pmol/mL) and decreased in the addicted group (43.21 pmol/mL) insignificantly compared to the control group (54.24 pmol/mL). 

**Fig.3:**
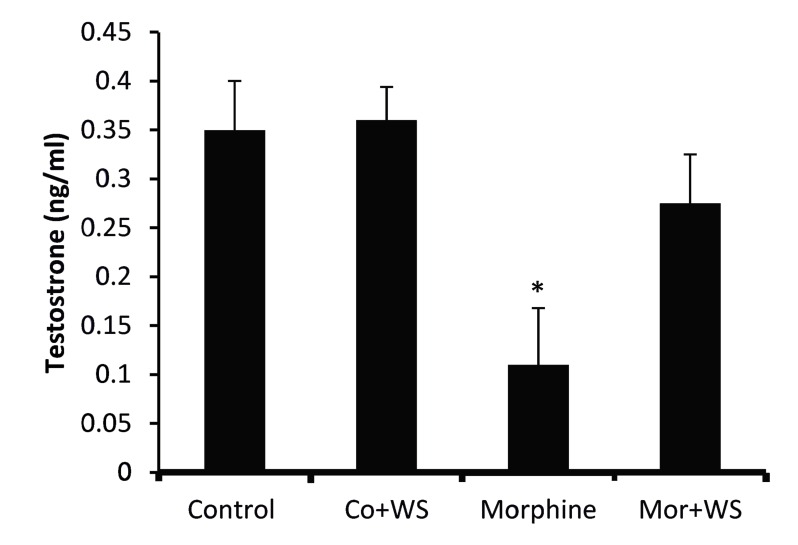
The effect of *Withania somnifera* root on testosterone levels in control and morphine addicted groups. Bars depict mean ± SEM. ^*^; Statistically significant, P<0.05 compared with the control and morphine addicted groups respectively.

Figure 3 shows that the testosterone level was decreased by morphine administration from 0.399 ng/mL to 0.112 ng/mL in the control group signifi- cantly (P<0.05). On the other hand, WS consump- tion in the addicted group inhibited the decrease in testosterone significantly (0.289 ng/mL, P<0.05)

**Fig.4:**
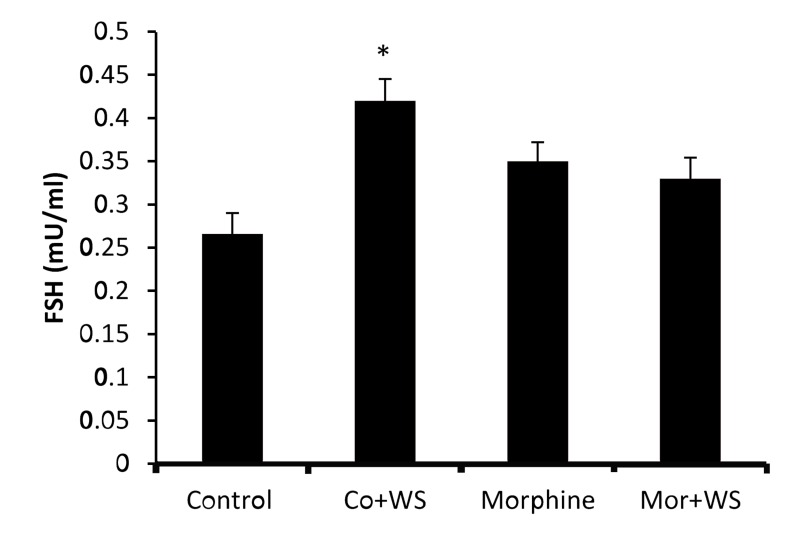
The effect of *Withania somnifera* root on FSH levels in control and morphine addicted groups. The data are expressed as mean ± SD. ^*^ ; P<0.05 as compared with the control group and FSH; Follicle-stimulating hormone.

The data on FSH are depicted in Figure 4, which shows that FSH had a statistically significant rise (P<0.05) only in the WS-treated control group (0.422 mIU/mL) compared to the control group (0.299 mIU/mL). 

**Fig.5:**
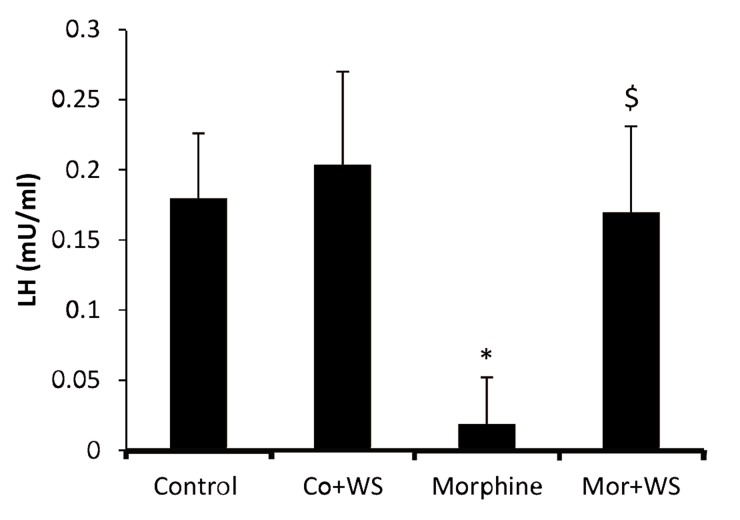
The effect of *Withania somnifera* root on LH levels in control and morphine addicted groups. The data are expressed as mean ± SD. ^*^, $; P<0.05 as compared with the control group and morphine addicted groups respectively and LH; Luteinizing hormone.

The data on LH levels in all the groups (Fig .5) revealed that there was a significant decrease in the LH level in the addicted male rats (0.0125 mIU/mL) compared to the control group (0.191 mIU/ mL). However, WS administration inhibited a decrease in the LH levels in the addicted rats significantly. 

## Discussion

In our male rats, morphine addiction significantly decreased testosterone and LH secretion, but not progesterone and FSH levels compared to the control group. Clinical studies have suggested that opiates may interfere with sex hormone secretion. Heroin use in men resulted in acute suppression of LH release from the pituitary followed by a secondary drop in plasma testosterone levels (21). Also, epidemiological studies have examined a possible link between hypogonadism and opioid use, in both patients and drug addicts. Statistically significant decreases in plasma hormone concentrations were found with lower testosterone and LH levels in men and lower estradiol, progesterone, LH, and FSH levels in women. Animal studies have provided consistent results (22). In male rats, chronic administration of morphine significantly decreased serum testosterone and LH levels, but not FSH release (23). The results of the present study are in agreement with the mentioned reports in human male addicts and male rats. 

It is suspected that opioids affect testosterone release through the hypothalamic-pituitary axis and inhibition of LH secretion (21,23). Opioids may decrease gonadotropins both by amending the sex hormone-hypothalamic feedback process and by interfering with the pituitary release of gonadotropins. Moreover, opioids can directly reduce testosterone secretion and testicular interstitial fluid by their negative effect on the testes (24). In the present study in addicted male rats, it seems that morphine caused suppression of LH release from the pituitary followed by a secondary drop in plasma testosterone levels. Therefore, it seems that morphine affects sex hormones and gonadotropins in male and female rats differently. 

In our animals-morphine decreased the estrogen level significantly, while the estrogen level was increased significantly in the WS-treated control group. In contrast, in the WS-treated addicted group, there was no decrease in the hormone. This finding chimes in with the result of another study in which the estrogen level was increased following the administration of WS (20). The same study also reported that WS might increase sexual steroids such as estrogen and progesterone via the stimulation of the hypothalamus-hypophysis axis. 

In the present study, morphine did not change the level of progesterone significantly. Therefore, in the morphine addicted groups, the WS root exerted no significant effect on progesterone levels. 

Our findings also revealed that a dietary intake of the WS root antagonized the reductive effect of morphine on testosterone and LH. Some studies have reported that some of the central effects of morphine are counteracted by the administration of the methanolic extract of the root of WS (25). Also, was shown that repeated administration of WS for 9 Rahmati 243 days attenuated the development of tolerance to the analgesic effect of morphine. WS also suppressed morphine-withdrawal jumps, a sign of the development of dependence on opiates (26). Accordingly, it seems that WS counters morphineinduced LH suppression release from the pituitary and subsequently enhances plasma testosterone levels. Elevated plasma testosterone levels by WS caused a secondary increase in estrogen levels. Since morphine did not change plasma FSH levels, WS also did not affect FSH levels in addicted male rats. On the other hand while LH and testosterone levels did not exhibit a significant change, FSH and estrogen levels increased significantly in the WS-treated control group. Therefore, it seems that WS affected sex hormones and gonadotropins differently depending on the treatment of the animals. Histological examinations have revealed an apparent increase in the diameter of seminiferous tubules and the number of seminiferous tubular cell layers in the testes of WStreated control immature rats (15). 

WS has been described in traditional medicine as an aphrodisiac that can be used to treat male sex dysfunction and infertility (27). It may, therefore, be suggested that in control normal rats, WS causes normal potentiation of the function of the testes by elevating FSH and its tropic effect on the Sertoli cells (15), without affecting testosterone as the principal male sex hormone. On the other hand, in addicted male rats with sex hormone and gonadotropin deficiency, WS recovered plasma LH and testosterone (23). Longterm treatment with the WS root extract resulted in a higher level of testosterone and LH among infertile men having suboptimal testosterone levels before therapy (27). Our findings in addicted male rats are concordant with the results obtained from infertile men having suboptimal testosterone levels. 

## Conclusion

In generally, sexual hormones and gonadotropin levels especially LH, testosterone, and estrogen were decreased by morphine administration. Nevertheless, WS administration in the addicted rats prevented the decrease in testosterone and LH. 
